# Supplementary figures and images for: Clinical manifestations and disease severity of SARS-CoV-2 infection among infants in Canada
Source: PLoS One. 2022 Aug 24;17(8):e0272648. doi: 10.1371/journal.pone.0272648 (PMC9401116; doi:10.1371/journal.pone.0272648)

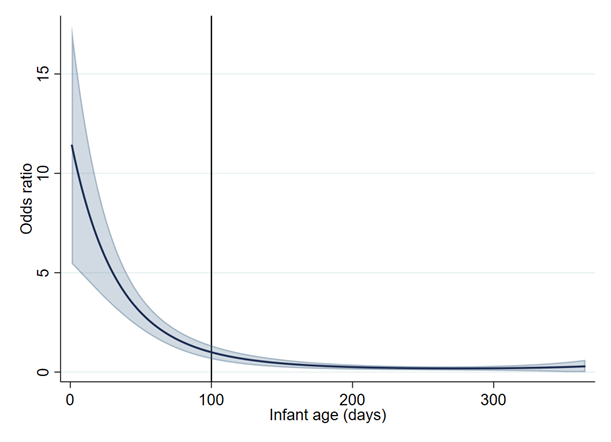

Supplement: S1 Fig — Odds ratios are adjusted by infant sex, gestational age category, comorbid conditions, and wave of pandemic. Continuous infant age was analyzed as a quadratic term. (TIF) [file pone.0272648.s001.tif]
