# Supplementary material for: Clinical manifestations and disease severity of SARS-CoV-2 infection among infants in Canada
Source: PLoS One. 2022 Aug 24;17(8):e0272648. doi: 10.1371/journal.pone.0272648 (PMC9401116; doi:10.1371/journal.pone.0272648)
Supplement: S1 Table — (PDF) [file pone.0272648.s002.pdf]

S1 Table. SARS-CoV-2 CPSP Severity criteria

Severity category	Clinical criteria	Outcome criteria
**Asymptomatic**	4.2: No symptoms reported, or write-in note specifying "asymptomatic"<br><br>4.4: No clinical features reported / no symptoms of COVID-19<br><br>OR<br><br>6.1, 6.2, 6.3: All presenting symptoms explained by concurrent infection	7.1: No targeted treatment against COVID-19 received<br><br>7.4: No respiratory support required<br><br>AND<br><br>Patient does not have any of the criteria for mild, moderate or severe disease.
**Outpatient care**	4.2: Any reported symptoms of COVID-19<br><br>4.4: Clinical syndromes may include gastrointestinal symptoms, skin changes, bronchiolitis or pneumonia	4.1. No admission required<br><br>7.1: No targeted treatment against COVID-19<br><br>7.3: Managed at home<br><br>7.4: No respiratory support required<br><br>AND<br><br>Patient does not have any of the criteria for moderate or severe disease.

**Mild disease**<br><br>**(in-patient)**	4.2: Reported symptoms may include fever, cough, sore throat, coryza, sneezing, lethargy (only for patients ≥1 month/≥1 year old), skin manifestations, muscle aches, rash, vomiting, diarrhea, loss of appetite, conjunctivitis, headache, loss of smell, lethargy, or loss of taste.<br><br>4.4: Clinical syndromes may include gastrointestinal symptoms, skin changes, bronchiolitis or pneumonia	4.1. Admission required<br><br>7.1: No targeted treatment against COVID-19<br><br>7.3: Inpatient ward<br><br>7.4: No respiratory support required<br><br>AND<br><br>Patient does not have any of the criteria for moderate or severe disease.
**Severity category**	**Clinical criteria**	**Outcome criteria**
**Moderate disease**	Any of the presenting symptoms described in the mild category	7.1: Targeted treatment against COVID-19, including Remdesivir, steroids, hydroxychloroquine/chloroquine, anti-IL1 or anti-IL6<br><br>7.4: Increased baseline home oxygen or low-flow oxygen<br><br>AND<br><br>Patient does not have any of the criteria for severe disease.

**Severe disease (any <u>one</u> of the listed criteria)**	4.2: Seizures, coma<br><br>4.4: Acute respiratory distress syndrome (ARDS), cytokine storm, seizures, stroke, encephalitis, encephalopathy, acute necrotizing encephalopathy, coma, hypotension, acute cardiac dysfunction	7.3: Intensive care unit admission<br><br>7.4: High-flow nasal cannula. Non-invasive ventilation (e.g., CPAP or BiPAP), Conventional mechanical ventilation, High-frequency oscillatory ventilation, Nitric oxide (NO), Extracorporeal membrane oxygenation (ECMO) or vasopressors.<br><br>7.6. Death (secondary to COVID-19)
