# Supplementary material for: Clinical manifestations and disease severity of SARS-CoV-2 infection among infants in Canada
Source: PLoS One. 2022 Aug 24;17(8):e0272648. doi: 10.1371/journal.pone.0272648 (PMC9401116; doi:10.1371/journal.pone.0272648)
Supplement: S2 Table — (PDF) [file pone.0272648.s003.pdf]

S2 Table. Symptoms, laboratory findings, and clinical syndromes of infants with SARS-CoV-2 infection by age group.

Clinical features	Infant age category		
	<1 month	1–12 months	*P* value[1]
**COVID-19-related hospitalizations, N[2]**	49	91	---
**COVID-19 severity, n (%)**			0.03
Asymptomatic/outpatient care	---	---	---
Inpatient care, mild/moderate	37 (75.5)	83 (91.2)	---
Inpatient care, severe	12 (24.5)	8 (8.8)	---
**Symptoms and signs, n (%)**			
Fever	36 (73.5)	70 (76.9)	0.65
Runny nose	21 (42.9)	35 (38.5)	---
Decreased oral intake	17 (34.7)	33 (36.3)	0.85
Respiratory distress	13 (26.5)	21 (23.1)	---
Cough	12 (24.5)	39 (42.9)	---
Lethargy	11 (22.4)	16 (17.6)	---
Sneezing	8 (16.3)	5 (5.5)	---
Vomiting	6 (12.2)	20 (22.0)	---
Diarrhea	7 (14.3)	16 (17.6)	---
Irritability[3]	5 (10.2)	8 (8.8)	---
Rash	<5 (<10.2)	6 (6.6)	---
Conjunctivitis	<5 (<10.2)	5 (5.5)	---
Tachycardia[3]	<5 (<10.2)	<5 (<5.5)	---
**Laboratory findings, n (%)**			
Neutropenia	5 (10.2)	16 (17.6)	---
Lymphopenia	<5 (<10.2)	12 (13.2)	---
Anemia	<5 (<10.2)	<5 (<5.5)	---
Thrombocytosis	0 (0.0)	6 (6.6)	---
**Clinical syndromes, n (%)**			
Upper respiratory tract infection	27 (55.1)	55 (60.4)	---
Gastrointestinal	14 (28.6)	30 (33.0)	---
Bronchiolitis	<5 (<10.2)	12 (13.2)	---
Pneumonia	<5 (<10.2)	8 (8.8)	---
Coagulopathy	<5 (<10.2)	<5 (<5.5)	---
Seizure(s)	<5 (<10.2)	<5 (<5.5)	---
Acute respiratory distress syndrome	<5 (<10.2)	0 (0.0)	---
Cardiac dysfunction	<5 (<10.2)	0 (0.0)	---
Hepatitis	0 (0.0)	<5 (<5.5)	---
**Abnormal CXR, n (%) / cases with imaging**	9/30 (30.0)	15/55 (27.3)	0.79
**Any respiratory support required, n (%)**	13 (26.5)	10 (11.0)	0.02
Low-flow oxygen	8 (16.3)	7 (7.7)	---
High-flow nasal cannula	<5 (<10.2)	<5 (<5.5)	---
Non-invasive ventilation	<5 (<10.2)	<5 (<5.5)	---

Mechanical ventilation[4]	<5 (<10.2)	<5 (<5.5)	---
Vasopressors	0 (0.0)	0 (0.0)	---
**Admitted to ICU, n (%)**	8 (16.3)	6 (6.6)	0.08
**Infant died, n (%)**	0 (0.0)	<5 (<5.5)	---

[1]Statistical tests conducted only for comparisons identified *a priori*.

[2]Excludes 58 hospitalizations not related to COVID-19, and one COVID-19-related hospitalization for whom an age category could not be assigned.

[3]Symptoms included as write-in notes only and may therefore be underrepresented.

[4]Includes conventional mechanical and high-frequency oscillatory ventilation.
