# Supplementary material for: Clinical manifestations and disease severity of SARS-CoV-2 infection among infants in Canada
Source: PLoS One. 2022 Aug 24;17(8):e0272648. doi: 10.1371/journal.pone.0272648 (PMC9401116; doi:10.1371/journal.pone.0272648)
Supplement: S3 Table — (PDF) [file pone.0272648.s004.pdf]

S3 Table. Logistic regression sensitivity analysis of factors associated with hospital admission due to COVID-19-related disease.

Characteristics	Odds ratio for COVID-19 hospitalization (hospitalizations unrelated to COVID-19 grouped with outpatient cases)				Odds ratio for COVID-19 hospitalization (restricted to all CHUSJ, HSC, and MCH cases[1])			
	Crude model		Adjusted model[2]		Crude model		Adjusted model[3]	
	OR (95% CI)	*P* value	aOR (95% CI)	*P* value	OR (95% CI)	*P* value	aOR (95% CI)	*P* value
**Infant age**								
0–<1 month	2.60 (1.49–4.52)	0.001	2.90 (1.62–5.17)	<0.001	3.89 (1.75–8.65)	0.001	3.41 (1.49–7.80)	0.004
1–3 months	1 [Reference]	NA	1 [Reference]	NA	1 [Reference]	NA	1 [Reference]	NA
4–6 months	0.32 (0.18–0.59)	<0.001	0.29 (0.15–0.54)	<0.001	0.42 (0.18–0.94)	0.035	0.30 (0.12–0.74)	0.009
7–12 months	0.18 (0.10–0.32)	<0.001	0.15 (0.08–0.29)	<0.001	0.25 (0.11–0.54)	<0.001	0.18 (0.07–0.42)	<0.001
**Infant sex**								
Female	1 [Reference]	NA	1 [Reference]	NA	1 [Reference]	NA	1 [Reference]	NA
Male	1.20 (0.81–1.77)	0.36	1.02 (0.65–1.59)	0.93	1.81 (1.03–3.20)	0.04	1.74 (0.91–3.33)	0.09
**Gestational age at birth**								
Term (≥37 weeks')	1 [Reference]	NA	1 [Reference]	NA	1 [Reference]	NA	1 [Reference]	NA
Late preterm (34–<37 weeks')	1.02 (0.44–2.36)	0.96	0.72 (0.28–1.87)	0.50	0.98 (0.27–3.58)	0.97	0.98 (0.22–4.27)	0.98
Moderate/very preterm (<34 weeks')	1.58 (0.70–3.54)	0.27	1.28 (0.51–3.20)	0.60	1.96 (0.57–6.72)	0.29	2.21 (0.55–8.95)	0.27
**Comorbid conditions**								
None/Unknown	1 [Reference]	NA	1 [Reference]	NA	1 [Reference]	NA	1 [Reference]	NA
≥1 comorbid condition	1.25 (0.69–2.25)	0.47	2.70 (1.32–5.53)	0.007	2.28 (1.01–5.13)	0.047	4.89 (1.79–13.35)	0.002
**Phase of COVID-19 pandemic**								
1st wave (April–August 2020)	1 [Reference]	NA	1 [Reference]	NA	1 [Reference]	NA	1 [Reference]	NA
2nd wave (September 2020–February 2021)	1.26 (0.70–2.27)	0.44	1.48 (0.77–2.85)	0.24	0.68 (0.33–1.42)	0.30	0.76 (0.33–1.73)	0.51
3rd wave (March–May 2021)	1.27 (0.68–2.38)	0.45	1.89 (0.93–3.82)	0.08	0.62 (0.28–1.36)	0.23	0.72 (0.29–1.80)	0.49

aOR = Adjusted odds ratio; OR = Odds ratio

[1]Centre Hospitalier Universitaire Sainte-Justine (Montreal), Hospital for Sick Children (Toronto), and Montreal Children's Hospital (Montreal).

[2]Multivariable analysis was conducted among 495 complete cases.

[3]Multivariable analysis was conducted among 317 complete cases.
