# Supplementary material for: Clinical manifestations and disease severity of SARS-CoV-2 infection among infants in Canada
Source: PLoS One. 2022 Aug 24;17(8):e0272648. doi: 10.1371/journal.pone.0272648 (PMC9401116; doi:10.1371/journal.pone.0272648)
Supplement: S4 Table — (PDF) [file pone.0272648.s005.pdf]

S4 Table. Characteristics of infants with SARS-CoV-2 infection reported from the Centre Hospitalier Universitaire Sainte-Justine, Hospital for Sick Children, or Montreal Children's Hospital.

Characteristics	All Infants	Outpatients	Inpatients		*P* value
			Not COVID-19 related	COVID-19 related	
**Total cases reported, N**	374	275	33	66	---
**Infant age (days), median (IQR)**	142 (52–253)	160 (73–263)	139 (52–262)	37 (22–115)	<0.001
**Infant age category, n (%)**					<0.001
<1 month	44 (11.8)	16 (5.8)	6 (18.2)	22 (33.3)	---
1–3 months	97 (25.9)	65 (23.6)	9 (27.3)	23 (34.8)	---
4–6 months	84 (22.5)	68 (24.7)	6 (18.2)	10 (15.2)	---
7–12 months	149 (39.8)	126 (45.8)	12 (36.4)	11 (16.7)	---
**Infant sex, n (%)**					0.11
Female	160 (42.8)	126 (45.8)	13 (39.4)	21 (31.8)	---
Male	214 (57.2)	149 (54.2)	20 (60.6)	45 (68.2)	---
**Population group of infant, n (%)[1]**					---
White	36 (9.6)	21 (7.6)	5 (15.2)	10 (15.2)	0.08
South Asian	43 (11.5)	27 (9.8)	5 (15.2)	11 (16.7)	0.21
Arab/West Asian	26 (7.0)	10–13 (3.6–4.7)	<5 (<15.2)	12–15 (18.2–22.7)	<0.001
Black	23 (6.1)	12 (4.4)	5 (15.2)	6 (9.1)	0.03
East/Southeast Asian	14 (3.7)	10 (3.6)	<5 (<15.2)	<5 (<7.6)	0.66
Indigenous	<5 (<1.3)	<5 (<1.8)	0 (0.0)	0 (0.0)	---
Other	11 (2.9)	7–10 (2.5–3.6)	<5 (<15.2)	0 (0.0)	>0.99
Unknown	228 (61.0)	188 (68.4)	13 (39.4)	27 (40.9)	<0.001
**Gestational age at birth, n (%)[2]**					<0.001
Term (≥37 weeks')	312 (89.1)	231 (92.0)	22 (66.7)	59 (89.4)	---
Late preterm (34–<37 weeks')	22 (6.3)	11–14 (4.4–5.6)	6–9 (18.2–27.3)	<5 (<7.6)	---
Moderate/very preterm (<34 weeks')	16 (4.6)	8 (3.2)	<5 (<15.2)	<5 (<7.6)	---
Median (IQR) weeks at birth[3]	34.6 (31.9–35.7)	34.4 (32.3–35.9)	34.6 (27.0–36.0)	31.9 (29.0–35.6)	0.62
**Any comorbid condition, n (%)**	44 (11.8)	20 (7.3)	14 (42.4)	10 (15.2)	<0.001
**Any co-infections, n (%)**	26 (7.0)	16 (5.8)	5 (15.2)	5 (7.6)	<0.001
**COVID-19 exposure, n (%)**					---

Known close contact with confirmed SARS-CoV-2 infection	211 (56.4)	146 (53.1)	14 (42.4)	51 (77.3)	<0.001
Nosocomial infection	<5 (<1.3)	0 (0.0)	<5 (<15.2)	0 (0.0)	<0.001
**SARS-CoV-2 lineage, n (%)**					
Cases before December 26, 2020[4]	145 (38.8)	102 (37.1)	15 (45.4)	28 (42.4)	---
Cases occurring after December 26, 2020	229 (61.2)	173 (62.9)	18 (54.6)	38 (57.6)	---
Alpha (B.1.1.7)	39 (17.0)	31 (17.9)	<5 (<27.8)	5–8 (13.2–21.1)	---
VOC, other or unspecified[5]	21 (9.2)	17 (9.8)	<5 (<27.8)	<5 (<13.2)	---
VOC not detected	35 (15.3)	29 (16.8)	0 (0.0)	6 (15.8)	---
VOC screening not conducted	134 (58.5)	96 (55.5)	15 (83.3)	23 (60.5)	---

[1]Physicians could report multiple population groups. East/Southeast Asian includes Chinese, Filipino, Japanese, Korean, and Southeast Asian. Indigenous includes First Nations, Inuit, and Métis.

[2]Gestational age category not known for 24 outpatients.

[3]Among preterm-born infants only (i.e. <37 weeks' gestation).

[4]i.e. Date of first Alpha detection in Canada.

[5]Includes B1351, P1, and variants with N501Y mutation but otherwise unspecified.
