# Supplementary material for: Clinical manifestations and disease severity of SARS-CoV-2 infection among infants in Canada
Source: PLoS One. 2022 Aug 24;17(8):e0272648. doi: 10.1371/journal.pone.0272648 (PMC9401116; doi:10.1371/journal.pone.0272648)
Supplement: S5 Table — (PDF) [file pone.0272648.s006.pdf]

**S5 Table. Comparison of CPSP and modified Dong severity criteria**

| CPSP classification | | Modified Dong classification | |
|---|---|---|---|
| Category | n (%) | Category | n (%) |
| Asymptomatic | 66 (12.4) | Asymptomatic | 66 (12.4) |
| Outpatient care | 310 (58.4) | Mild disease | 334 (62.9) |
| Inpatient - mild disease | 125 (23.5) | Moderate disease | 79 (14.9) |
| Inpatient - moderate disease | 10 (1.9) | Severe disease | 28 (5.3) |
| Inpatient - severe disease | 20 (3.8) | Critical disease | 24 (4.5) |
