# Supplementary material for: Clinical manifestations and disease severity of SARS-CoV-2 infection among infants in Canada
Source: PLoS One. 2022 Aug 24;17(8):e0272648. doi: 10.1371/journal.pone.0272648 (PMC9401116; doi:10.1371/journal.pone.0272648)
Supplement: S6 Table — (PDF) [file pone.0272648.s007.pdf]

S6 Table. Characteristics of admitted infants with severe COVID-19

Characteristics, n (%)	Frequency	Percent
**Admissions with severe COVID-19, N**	20	---
**Admitted to ICU**	14	70.0
**Any respiratory/hemodynamic support required**	15	75.0
Low-flow oxygen	7	35.0
High-flow nasal cannula	5	25.0
Non-invasive ventilation	<5	<25.0
Mechanical ventilation[1]	5	25.0
Vasopressors	0	0.0
**Clinical syndromes**		
URTI	8	40.0
Pneumonia	5	25.0
Acute cardiac dysfunction	<5	<25.0
Acute respiratory distress syndrome	<5	<25.0
Bronchiolitis	<5	<25.0
Encephalopathy	<5	<25.0
Gastrointestinal	<5	<25.0
Hematologic disorder[2]	<5	<25.0
Seizure(s)	<5	<25.0
**Radiologic findings**		
Abnormal chest x-ray	8	40.0
Abnormal CT scan	0	0.0
**Infant died**	<5	<25.0

URTI=Upper respiratory tract infection.

[1]Includes conventional mechanical and high-frequency oscillatory ventilation.

[2]Includes anemia, lymphopenia, neutropenia, and thrombocytosis.
