# Supplementary material for: Clinical manifestations and disease severity of SARS-CoV-2 infection among infants in Canada
Source: PLoS One. 2022 Aug 24;17(8):e0272648. doi: 10.1371/journal.pone.0272648 (PMC9401116; doi:10.1371/journal.pone.0272648)
Supplement: S7 Table — (PDF) [file pone.0272648.s008.pdf]

S7 Table. Treatments administered to infants with COVID-19

Characteristics, n (%)	Frequency	Patient group			Child age			
		Outpatients	Inpatient, COVID-19-related	P value	<1 month	1–3 months	4–12 months	P value
**Total patients, N (excludes hospitalizations not related to COVID-19)[1]**	473	332	141	---	69	138	262	---
**Prescribed any treatment**				<0.001				<0.001
Yes	98 (20.7)	18 (5.4)	80 (56.7)	---	35 (50.7)	34 (24.6)	29 (11.1)	---
No	375 (79.3)	314 (94.6)	61 (43.3)	---	34 (49.3)	104 (75.4)	233 (88.9)	---
**Treatments received**								
Antibioitics	94 (19.9)	16 (4.8)	78 (55.3)	<0.001	35 (50.7)	34 (24.6)	25 (9.5)	<0.001
Steroids	11 (2.3)	---	---	---	---	---	---	---
Immunoglobulin (IVIG)	<5 (<1.1)	---	---	---	---	---	---	---
Remdesivir	<5 (<1.1)	---	---	---	---	---	---	---
Other[2]	6 (1.3)	---	---	---	---	---	---	---

[1]N=58. Age category not available for 4 infants.

[2]Includes antivirals other than remdesivir, aspirin, and bronchodilators.
