# Supplementary material for: Clinical manifestations and disease severity of SARS-CoV-2 infection among infants in Canada
Source: PLoS One. 2022 Aug 24;17(8):e0272648. doi: 10.1371/journal.pone.0272648 (PMC9401116; doi:10.1371/journal.pone.0272648)
Supplement: Attachment — Submitted filename: reviewerresponse.docx [file pone.0272648.s009.docx]

Reviewer #1: This is a well written paper that provides a nice description of infants and their manifestations from COVID-19. I only have a two major thread of comments. The issue is the selection approach is not clear. The results from the selection approach are biased as well (connected to the patient selection approach), and this need to be clarified. These comments are specifically outlined below.

*We with to thank the reviewer for the comments and feedback on our manuscript. Below are our responses to the detailed comments. We are hoping that the reviewer will find that the updated manuscript has appropriately answered all of their queries.*

**Abstract**
1. Page 3, line 69: 37.5% admission clearly is biased if you are looking at this as all infants that acquired COVID-19. I think its important to make this clear throughout the paper. For instance on page 4, line 78, the authors state that most are mildly ill, but this doesn't compute with the 35% admission rate. The denominator of asymptomatic and not sick infants is not part of the study, so you need to be clear about this.

*The abstract conclusion was modified to highlight that severe COVID-19 was found to be rare (even amongst hospitalized cases) and the wording on mild disease was removed. Cases of asymptomatic SARS-CoV-2 infection could be included in the study, however, there may indeed be selection bias and under-reporting as described in the study limitation.*

**METHODS**
2. Page 6, line 109: The CPSP needs to be clarified. Are these outpatient clinics? How does the hospital enrollment overlap or not?

*Pediatricians are members of the CPSP independent of their practice location, may it be in working in hospital settings or outpatient clinics. The pediatricians themselves form the surveillance network rather than the clinics or hospitals. This was clarified in the methods.*

3. Clarify that you did not include asymptomatic and those without significant disease from the CPSP, since it appears you did not. Or if I am not understanding this, please clarify.

*Asymptomatic cases could be reported and were classified separately under the severity classification (for example, as reported in Table 3). This was clarified in the methods and is also reported in the* ***Study definitions*** *section (under the definition for asymptomatic cases).*

4. Page 7, lines 136 - 139: It appears you have included 2800 outpatient practices and then also added 3 hospitals. Are the same participants included in both? If so, was that to obtain the outpatient + inpatient course together? Or are these separate? The three categories appears to me to be (1) CPSP, (2) either CPSP or hospital, (3) hospitals. I am not sure if this is right and it matters since it impacts your results.

*The CPSP is a network of 2,800* ***pediatricians*** *who are working in both outpatient and hospital settings. The three centers that were mentioned are part of the CPSP in that CPSP pediatricians are working in these institutions, including the main study investigators. The difference with other settings was the sampling strategy of active case finding using EMR system to systematically collect data on infants, which was not implemented in other settings. Cases could only be reported once, and duplicate data was removed. This was specified in the manuscript.*

**RESULTS**
5. Clarify the results with the selection criteria so its more clear how the results are connected (or not).

*As detailed in the methods, all cases of SARS-CoV-2 in infants aged less than one year old who were seen by pediatricians from the CPSP were eligible for inclusion, if they were reported to the network. All microbiologically-confirmed cases of SARS-CoV-2 that were reported to CPSP were included in the analyses.*

6. Page 15, lines 253 - 256 -- The criteria for hospitalization in young infants is pretty large -- fever in particular. While this is noted in the Conclusions, the results seem driven by this admission criteria and it should be pointed out sooner.

*A line to that effect was added to the manuscript, highlighting the percentage of infants <1 month of age who experienced fever.*

7. Page 15, line 257 -- Please also give the age in months since it seems odd to flip from one to the other.

*Age was modified from days to months throughout the manuscript.*

8. Page 19, line 280: The fact that 78.7% of admitted children had mild disease shows the bias based on the selection approach and also the admission criteria, and should not be considered a finding of how sick these children are.

*As detailed in the methods, all cases of SARS-CoV-2 in infants aged less than one year old who were reported to CPSP were included, without further selection of cases. The study aimed to capture all cases that met the inclusion criteria, as detailed in the methods section. Criteria for hospital admission were entirely clinical, made by clinicians, and there were no study-directed decisions or criteria about hospital admission. The reported percentage of 78.7% represents the percentage of all infants admitted for COVID-19 who had mild disease, based on the pre-established criteria for severity.*

**DISCUSSION**
9. Page 23, second paragraph -- I do not believe the authors showed that COVID-19 is uncommon in children with this paper.

*We agree with the reviewer that the study has not shown that COVID-19 was uncommon in infants. This paragraph states that* ***severe*** *COVID-19 was uncommon within all cases that have been reported (3.8%).*
